# Highly stretchable sensing array for independent detection of pressure and strain exploiting structural and resistive control

**DOI:** 10.1038/s41598-020-69689-2

**Published:** 2020-07-29

**Authors:** Ryosuke Matsuda, Satoru Mizuguchi, Fumika Nakamura, Takuma Endo, Yutaka Isoda, Go Inamori, Hiroki Ota

**Affiliations:** 1 Department of Mechanical Engineering, Yokohama National University, 79-5 Tokiwadai, Hodogaya-ku, Yokohama, 240-8501 Japan; 2 Graduate School of System Integration, Yokohama National University, 79-5 Tokiwadai, Hodogaya-ku, Yokohama, 240-8501 Japan

**Keywords:** Electrical and electronic engineering, Polymers

## Abstract

Stretchable physical sensors are crucial for the development of advanced electrical systems, particularly wearable devices and soft robotics. Currently available stretchable sensors that detect both pressure and strain are based on piezoelectric, piezoresistive, or piezocapacitive effects. The range of pressure sensing is 1–800 kPa with large deformations being within the range of deformations of parts of the human body, such as elbows and knees. However, these devices cannot easily allow simultaneous and independent detection of pressure and strain with sensor arrays at large tensions (> 50%) because strain affects the pressure signal. In this study, we propose a monolithic silicone-based array of pressure and strain sensors that can simultaneously and independently detect the in-plane biaxial tensile deformation and pressure. To realize these functionalities, the deformation of the device structure was optimized using a hetero-silicone substrate made of two types of silicone with different hardness characteristics and porous silicone bodies. In addition, the resistances of the sensors were controlled by adjusting a mixture based on carbon nanoparticles to improve the sensitivity and independence between the pressure and strain sensors. These concepts demonstrate the potential of this approach and its compatibility with the current architectures of stretchable physical sensors.

## Introduction

Stretchable sensors are in high demand for electronics used in advanced electric systems^1–7^ particularly for wearable devices. Several stretchable physical sensors have been developed, including pressure^8–10^, strain^10–13^, temperature^14,15^, shear force^16^, and light^17,18^ to detect human motion^19^ or condition^20–22^. Many multi-physical sensors with stretchability have been developed for combined detection of these physical parameters^23–25^. Among stretchable devices, pressure and strain sensors are two of the most fundamental and important devices for the evaluation of the physical characteristics of body deformations and human movements. The benchmark for stretchability in terms of wearable devices is ~ 50% to match the maximum deformations of parts of the human body^26^, such as elbows and knees.

Available stretchable pressure sensors are constructed based on piezoresistive, piezocapacitive, and piezoelectric effects. These pressure sensors are composed of a liquid material for wiring^27,28^ using complex-coated carbon nanotubes^23^ and porous silicone^29^. These devices can detect the applied pressure with high stretchability and are applied to electric skins in wearable devices. Currently, stretchable pressure sensors produce pressure measurement errors when subjected to strain deformation that hinders the detection of pressure and strain simultaneously and independently.

A pressure sensor that can detect strain at the same time was realized using a liquid metal-based pressure sensor^24^. The strain and pressure sensors inside the device were switched according to the values of each sensor: when the device receives an x-axis stimulus, only the x-axis strain sensor is activated. Another pressure and strain sensor composed of carbon nano tube^23^ and silver nanowires^24^ can measure both pressure and strain. These devices measure pressure and strain using the same mechanism, when the device is stretched or pressed, the distance between the upper and lower layers decreases. However, sensing arrays that can detect both pressure (over a large range of values) and strain (> 50%) have not yet been implemented. In particular, it is still difficult to detect the resistance or capacitance of each pressure sensor independently during large deformations. To ensure the detection of multiple physical deformations by humans, devices on the body surface should recognize and detect such deformations individually and within large ranges of pressure and tension (> 50%).

In this paper, we propose a sensing array that can detect pressure from an upper surface and large strains (> 50%) along the x/y axis simultaneously and independently. The detection of pressure and strain is based on the resistance properties of carbon nanoparticles. The resistances of the sensor and electrodes were optimized by adjusting the mixture of carbon nanoparticles and fluoropolymer to independently detect the resistance variation using each pressure and strain sensor in an array and minimize the mutual interference between tension and pressure signals owing to deformations. Furthermore, the resistance variation of the pressure sensor was controlled structurally by a hetero-silicone substrate composed of hard polydimethylsliloxane (PDMS) and soft Ecoflex, as well as porous carbon-coated silicone. The components of the device are made of highly biocompatible materials. This is crucial for the development of wearable physical sensors.

## Results

### Device design and fabrication

**Figure 1:**
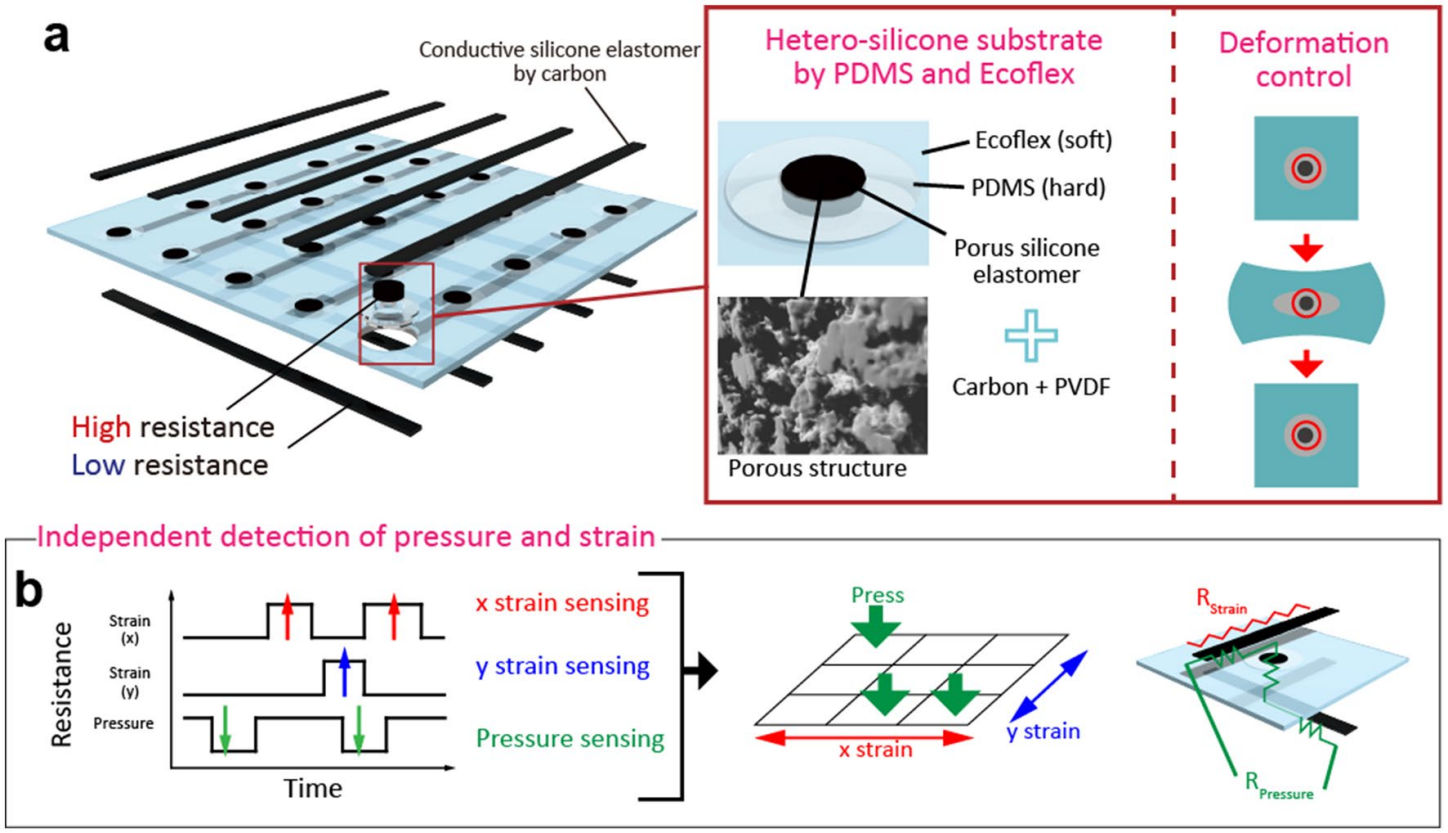
Stretchable pressure sensing array for independent detection of x and y tensions. (**a**) Schematic of the array. This silicone substrate is made of two different silicone types with different hardness values. Harder silicone, PDMS, can suppress the deformation of pressure sensing elements during tension. (**b**) Schematic of measurement methods and signals of a pressure mapping sensor subjected to strain deformation. This sensor can independently sense three different stimuli, namely pressure, x-, and y-directional strains.

As shown in Fig. 1a, soft porous silicone coated with a composite of Super P and polyvinylidene difluoride (PVDF) was used as the pressure sensor. The sensor enabled pressure detection based on the piezoresistive effect. The standard resistance value of the sensing element can be controlled by changing the properties of the conductive liquid (e.g., liquid metal) in the porous silicone. The hetero-silicone substrate was composed of hard silicone (PDMS) that formed the pressure sensors and soft silicone (Ecoflex). The PDMS around the pressure sensing elements prevents the development of large deformations of the elements during the developed device tension. This suppresses the resistance variation of the pressure sensor induced by strain. The conductive paste was composed of Super P carbon with high elasticity and was deposited on the top and bottom layers to achieve x and y strain sensors based on resistance variations along the x–y axis.

The circuit of each sensor was different to independently detect mapping of the pressure and strain of the x and y directions. Strain sensing is achieved using a conductive silicone elastomer consisting of carbon nanoparticles and Ecoflex on the top or bottom layers, as shown in Fig. 1b. On the other hand, pressure sensing is achieved using a porous silicone elastomer via the conductive silicone elastomers on the top and bottom layers. However, the resistivity of the porous conductive silicone elastomer is significantly higher than that of the conductive silicone elastomers. Therefore, pressure and sensing of the x/y strain can be detected independently. To generate sensor maps, we constructed the passive matrix structure using the arrangement of patterned porous silicone in an array of pressure sensor elements based on the description of lines of stretchable conductive silicone paste on the top and bottom layers of the array.

**Figure 2:**
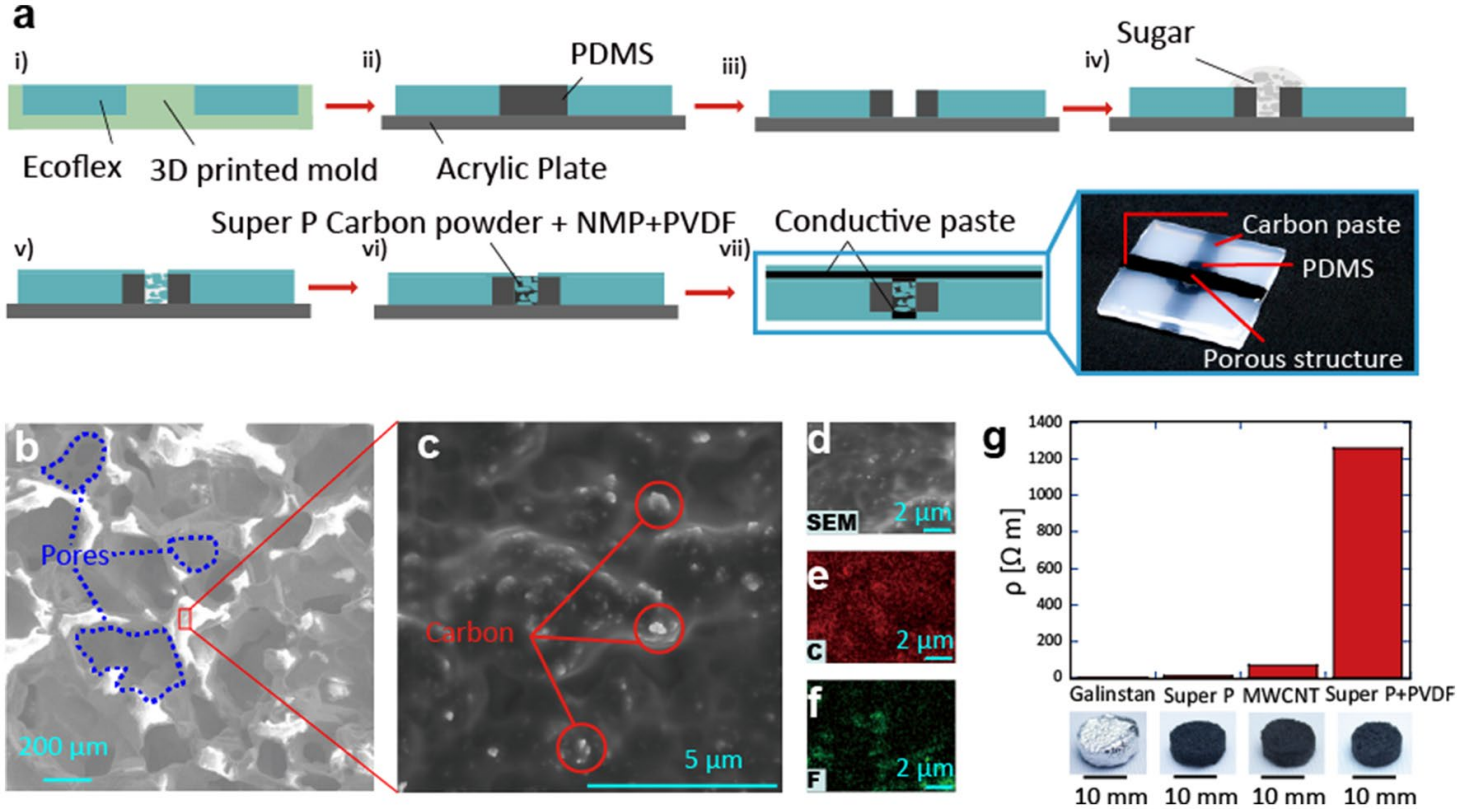
Fabrication process and characteristics of a single-pixel device. (**a**) Fabrication process. (**b**,**c**) Scanning electron microscopy (SEM) image of porous pressure sensing element infiltrated by a carbon/PVDF mixture. Higher magnification SEM image of the porous silicone. (**d**–**f**) SEM and energy dispersive spectroscopy (EDS) analytics of carbon and fluorine in the pressure sensing element. (**g**) Resistance differences according to the conductive material in the porous silicone.

Figure 2a shows the fabrication process of a single-pixel device. First, liquid Ecoflex 00-20 was poured onto a three-dimensional (3D) mold (i). After curing and peeling off the Ecoflex substrate from the mold, liquid PDMS was poured into a hole in the substrate (ii). After curing PDMS, a smaller hole was punched to allow placement of the pressure sensor (iii). Sugar was added into the hole to create pores inside Ecoflex. Liquid Ecoflex was then poured into this part. By vacuuming the substrate, Ecoflex penetrated the regions in which sugar was deposited. After curing Ecoflex, the substrate was sonicated through ultrasonication to dissolve sugar (v). A solution of Super P carbon, fluoropolymer, PVDF, and *N*-methylpyrrolidone (NMP) was poured into the porous Ecoflex and penetrated this part (vi). This porous structure increased the resistance of the pressure sensing part. Finally, the column and row electrodes of the carbon paste were formed on the top and bottom sides of the substrate for the detection of the x and y strains. Acquisition of highly magnified images and analysis of molecules was performed using scanning electron microscopy (SEM) and with energy dispersive X-ray spectroscopy (EDS) of the carbon and fluorine elements (Fig. 2b–f). The resistance of the porous silicone pressure sensing element was different, depending on the material used in coating the surface of the porous silicone (Fig. 2g).

### Testing and results

**Figure 3:**
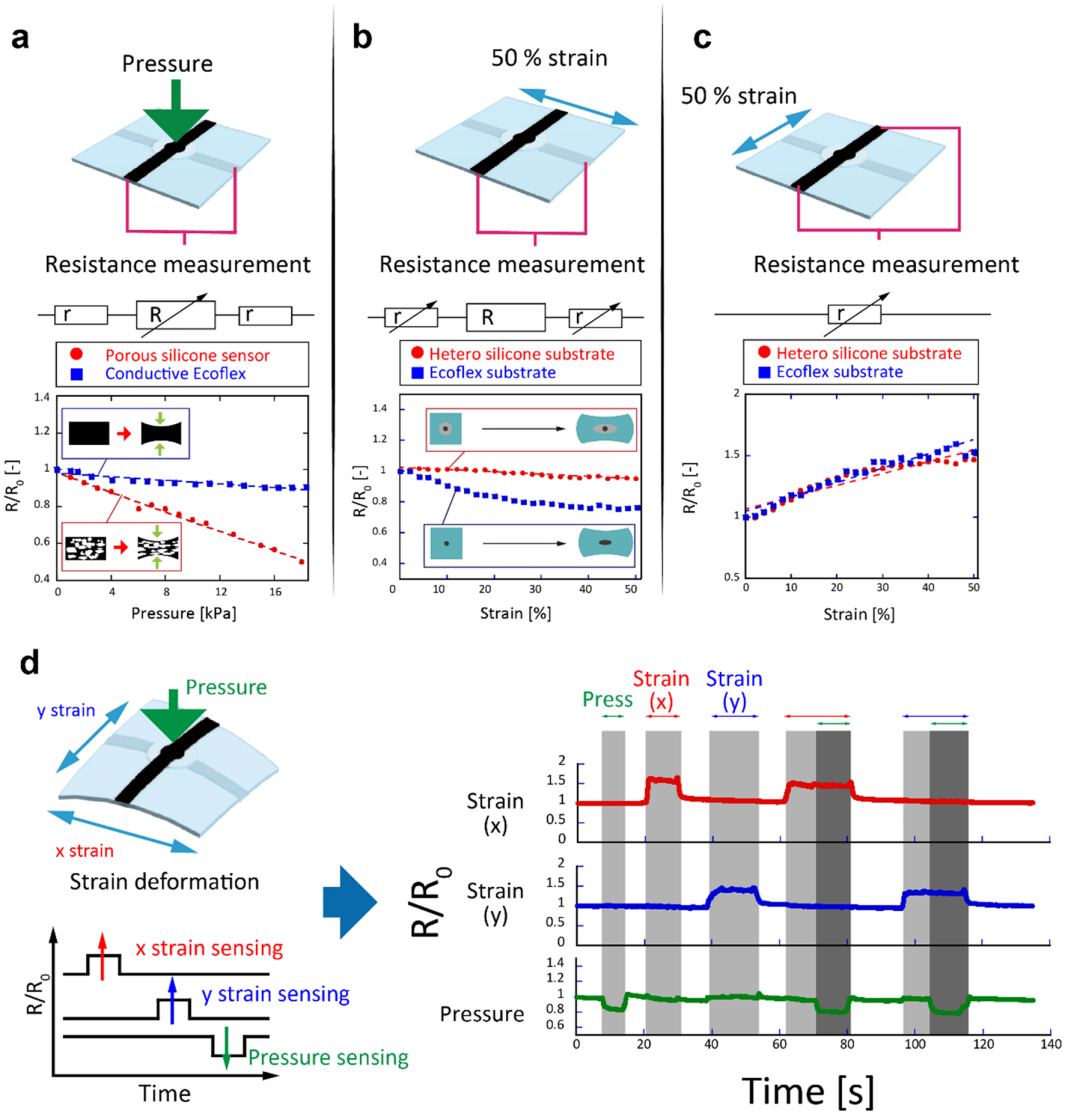
Sensing characteristics of a single-pixel device. (**a**) Resistance variation of a pressure sensor composed of porous and conductive silicone with respect to pressure. Resistance “r” in this figure is low and resistance “R” is high. (**b**) Resistance variation of pressure sensors on the entire Ecoflex and hetero-substrates subjected to 50% strain deformation. (**c**) Resistance increases with respect to strain deformation. (**d**) Demonstration of independent pressure and x and y strain detection using a single-pixel device. The resistance along the x and y axes increased while this device was strained in the x and y directions. In contrast, the pressure sensor resistance decreased while the sensor of the device was pressed. In this demonstration, the pressure and strain stimuli were sensed independently while the stimuli were applied simultaneously.

The results of the pressure and strain experiments conducted with the single-pixel device are shown in Fig. 3. The resistance of the pressure sensors was measured based on the electrical path of the porous conductive silicone and conductive paste on the top and bottom layers. The resistivity of the porous conductive silicone was 1,000 times higher than that of the conductive silicone in the strain sensor, and strain sensor’s resistance was enough small to be neglected while detecting the pressure. Both the x- and y-axis electrodes are needed to measure pressure. As shown in Supplementary Fig. S4, the y-axis strain was not significantly affected by x-axis tensile deformation (< 1.3% during 50% strain at the other axis) because the cross sectional area of the strain sensor might not be affected by stretching. That is, even if the height of the cross section becomes small, the width would be large. The resistance of the porous conductive silicone decreased with respect to pressure, and its sensitivity was much higher than that of the nonporous conductive silicone composed of the same materials. The porous silicone used in this device had high flexibility compared with normal Ecoflex. In fact, the Young's modulus was ~ 5 kPa, which is seven times lower than that of regular Ecoflex (see Supplementary Fig. S1). These results show that the porous silicone can be used to increase the sensitivity of the pressure sensor. Figure 3b shows the stability of the resistance of the pressure elements when subjected to a 50% tension compared with hetero-silicone and Ecoflex substrates. The resistance of a device that uses a hetero-silicone substrate barely changes even when the device is subjected to 50% tension.

In contrast, the resistance of a device whose substrate was only composed of Ecoflex decreased to approximately 70% when the device was subjected to 50% tension. As mentioned earlier, the resistance of the circuit was dominated by the pressure element. The change in the area of the pressure sensing part was only 1.7%, as shown in Supplementary Fig. S2. This explains why the resistance of the pressure sensor in the device did not change when the device was subjected to 50% tension. Figure 3c shows the resistance change of the conductive silicone line on the top layer of the device during the period in which the device was subjected to 50% tension. The resistance of the strain sensor increased with respect to the strain in both devices, that is, in the device composed of hetero-silicone substrate and the device composed of Ecoflex substrate. Both devices possess almost the same sensitivities, and the resistance was stable after the application of 100 cycles of strain (with the device subjected to 50% strain at each cycle) (see Supplementary Fig. S3). Figure 3d shows the resistance changes associated with the pressure and x and y strain sensors when a pressure of 5 kPa and 50% tension were applied. The device detected biaxial tension and pressure independently. The pressure sensor exhibited an error of 1 kPa when the device was subjected to 50% strain. This error is considered to be very small, particularly for device strains lower than 50%. During tension, the device can become thinner. However, the solid silicone and PDMS around the pressure sensing part can suppress deformation of the part in the perpendicular direction. Therefore, pressure sensing was not affected by large tension in the devices. The standard resistance value depends highly on the structures and materials of the sensor components, as shown in Supplementary Fig. S5. In particular, the real resistance value of the pressure sensors was more than 10 times higher than that of the strain sensors, suppressing the effect of tension on the pressure sensor. The resistance value can be adjusted depending on the application and structure.

**Figure 4:**
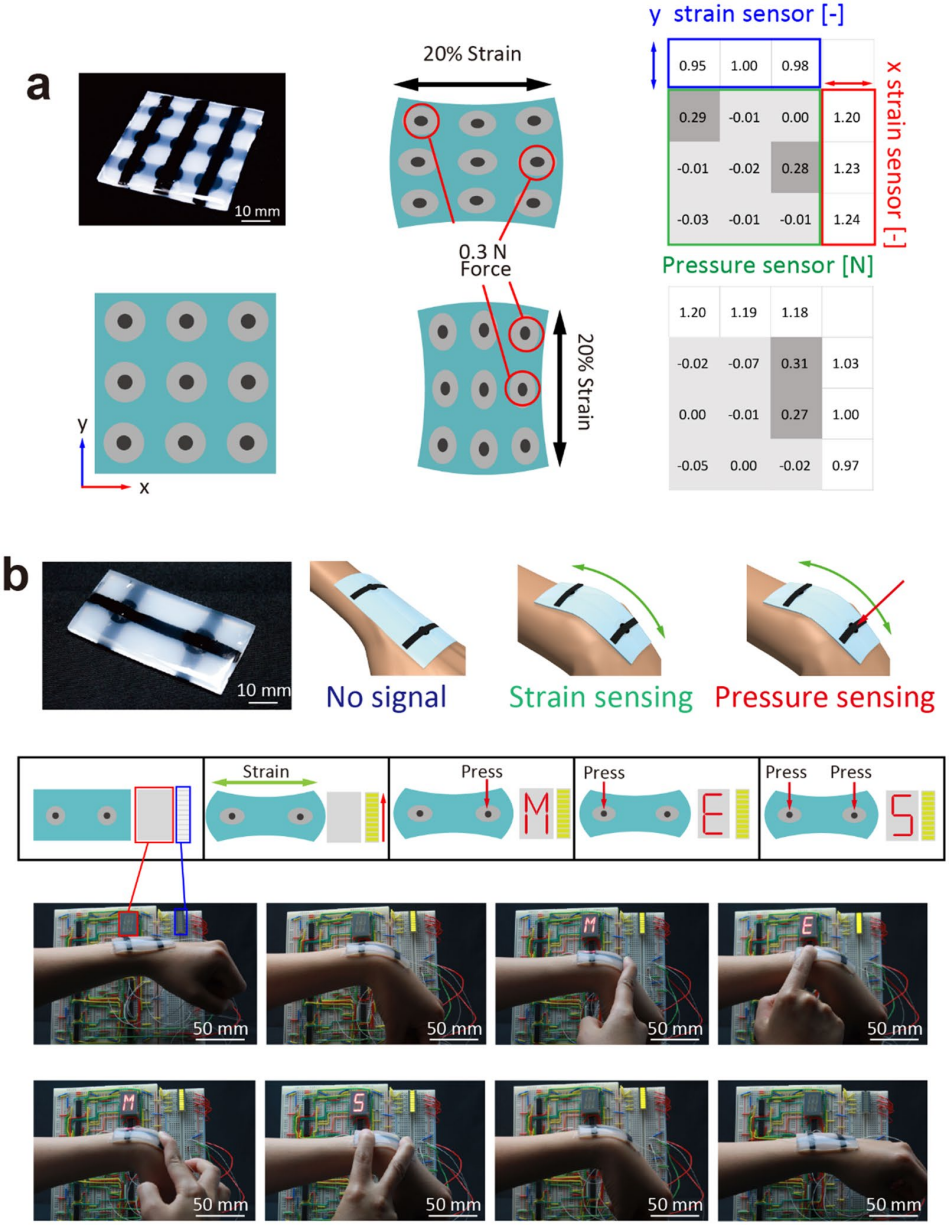
Demonstration of functionality of a multipixel device. (**a**) Maps of pressure and strain using the 9-pixel device. The device achieved 9-pixel mapping of pressure when subjected to large strains. In addition, it sensed strain using six conductive lines based on a passive matrix. (**b**) Demonstration of display controlled by a 2-pixel device with a strain indicator. The digital display controlled by the 2-pixel device shows the word “MEMS” and works like a keyboard. In addition, the strain indicator, which was composed of an LED bar, was turned based on the amount of strain. Pressure and strain sensing were independently controlled by each motion.

We extended the design to 9 pixels for mapping of pressure and x and y strain sensing based on accumulated experience with the single-pixel device. Figure 4a shows a demonstration of the mapping process when a force of 0.3 N was applied at an arbitrary point while the device was subjected to strain. The positions and the amount of the applied pressure were detected, while the strain deformation was ignored. In addition, the tensile deformations of the device were also detected based on measurements of the resistance changes of the strain sensors. In the analysis of the applied pressures of this device, the resistance of the desired position in the map was affected by different electric paths (Supplementary Information 1). This explains why calculations were performed based on formulated equations from each electrical pathway to measure the precise resistance of the pressures at each point. In this demonstration, 20% strain was used because the area occupied by the PDMS of the 9-pixel device was larger compared with the device size (see Supplementary Fig. S12). The device itself can be extended by 50%; however, it was difficult to extend the center of the device by 50%. On the other hand, it was possible to apply 50% strain to a 4-pixel device having the same ratio of the PDMS part as the devices in Fig. 3 in the same demonstration (see Supplementary Fig. S13). It may be necessary to optimize the design of the device depending on the application.

Figure 4b shows a demonstration of the display control based on a keyboard configuration using a 2-pixel device on a human wrist. This device was equipped with two porous silicone structures used as pressure sensors and one conductive silicone line used as the strain sensor. The device was attached to a wrist. As shown in Fig. 4b and Supplementary Video S1, the light emitting diode (LED) bar is lit in five stages according to the bending of the wrist. This means that the device attached to the wrist detected the strain of the device during the bending process. In addition to the strain motion, the digital display system displayed the letters “M,” “E,” and “S,” on a 16-segment LED display according to the combination of input signals from two pressure sensors on the device. The pressure sensors were not affected by this bending deformation. Therefore, no character was displayed on the LED monitor upon bending. In contrast, lighting of the LED light bar as a result of wrist flexion was not attenuated by the application of pressure. This indicates that strain and pressure were detected independently using the developed device.

## Discussion

In the fabrication process used for the pressure sensors in our device, caster sugar was used to synthesize the porous PDMS structure instead of granulated sugar commonly used in the fabrication process. Granulated sugar consists of particles with diameters in the range of 300–500 µm, whereas caster sugar is composed of particles with diameters in the range of 100–200 µm. SEM images of porous silicone fabricated with caster or granulated sugar are shown in Supplementary Fig. S6. The robustness of the porous silicone was altered depending on the pore size. That is, the strength of the porous silicone increased with respect to the pore size. In this study, caster sugar was used to fabricate the porous structure to obtain increased robustness because the deformation induced by the developed tension should be suppressed. The particle size of the sugar is approximately 200 µm. This is the reason the device requires a thickness of 2 mm to create a sufficient porous network using caster sugar. Polystyrene beads^30^ and polymethyl methacrylate (PMMA)^31^ can be used instead of caster sugar to decrease the thickness for practical use of the device. The processing method of the pore structure composed of smaller holes using these materials have been demonstrated in other studies^30,31^. The use of a smaller size of the particle can decrease the device thickness.

This study utilized the advantages of Super P carbon black used as conductive material inside the porous silicone. The mixture of carbon nanotubes (CNT) and Super P carbon proved useful for adjusting the deformation sensitivity. The device used in this study required increased strain sensor sensitivity. Therefore, Super P carbon was adopted^32^.

In terms of the device construction, the sensitivity of the sensor with porous body was 4.8% kPa^−1^, and that with regular conductive silicone was 0.6% kPa^−1^ (see Supplementary Fig. S7). The sensitivity of the porous silicone-based pressure sensor was 8 times higher than that composed of an entire conductive Ecoflex sensor, as shown in Supplementary Fig. S7. The resistance of the porous silicone can be controlled based on the amount of carbon and the ratio of PVDF to achieve values in the range of 1.3–4,260 kΩ (Supplementary Figs. S8, S9). In addition, Supplementary Figs. S10 and S11 show that the resistances of the conductive sponge and paste increased linearly as a function of their length. Therefore, the ratio of PVDF and the amount of carbon particle should be appropriately selected to optimize the device construction depending on the application.

Supplementary Table S1 presents a comparison of the stretchable pressure sensors used in this study and in other studies. The sensitivity was 1,000% kPa^−1^ for a highly sensitive stretchable pressure mapping sensor using a conductive material and microstructure^33,34^ (see Supplementary Table S1, items i, ii). These studies reported excellent pressure sensitivities. On the other hand, the ranges of the linear current variations of these sensors are narrow, and the sensitivities vary depending on the applied pressure. In addition, the pressure sensor with conductive stretchable polymer yielded a resistance change of approximately 30% for 33% tension^34^ (see Supplementary Table S1, item ii). The developed device had a broad detectable range because the resistance decreased linearly with respect to pressure up to approximately 18 kPa (Fig. 3a). Compared with liquid metal-based pressure sensors with high stretchability and conductive stability^27^, the sensitivity of our device was more than two times that of a liquid metal sensor with a diameter of 15 mm (see Supplementary Table S1, item iii).

Supplementary Table S1 highlights the limitation of tensile deformation without any effects on pressure sensing, in which the fabricated device in this study could be stretched up to 50% by ignoring strain-induced deformations (see Supplementary Table S1, item viii). This result demonstrates the significant benefit of the hetero-silicone substrate structure. In another study, the capacitance variation of the pressure sensor was approximately 20%^23^ (see Supplementary Table S1, item iv) and 35%^24^ (see Supplementary Table S1, item v) when 50% tension was applied, whereas in yet another study, the resistance variation was 600%^25^ when 45% tension was applied (see Supplementary Table S1, item vi).

In these studies, it may be difficult to recognize pressure and strain in a signal device given that signals from pressure and strain affect each other. In contrast, stretchable pressure sensors that used metal thin film demonstrated stable pressure sensing even when 15% strains were applied^35^ (see Supplementary Table S1, item vii). These sensors possess high-pressure sensing sensitivity but did not exhibit stable pressure sensing during large deformations (more than 20%). The device with hetero-silicone substrate developed in this study yielded a maximum resistance variation of 4.9% when subjected to 50% strain deformation. This is equal to the variation obtained when a pressure of 1 kPa was applied in the pressure test, as shown in Fig. 3a.

In summary, the developed device can independently detect pressure at large deformations (up to 50%) by exploiting structural and resistive control. In this study, the resistivity of stretchable conductive components was controlled from 1 to 1,000 Ω·m using the fine porous structure and the mixture of carbon nanomaterials. It is crucial to assemble electrical components for stretchable devices such as electrodes and sensors. In addition, the device utilized the advantage of the hetero-silicone substrate to prevent strain from affecting the pressure element responses. As a result, a combination of these structural optimization and resistive control schemes of the device assembly achieved a stretchable array of pressure and strain sensing within large ranges independently. Independent detection of tensile deformation and pressure is important in fields in which complex pressure patterns are applied during large deformations. These developed devices might be useful as wearable devices and surface mounted sensors on soft robots and actuators associated with large deformations.

## Methods

### Preparation of conductive materials

#### High resistance solution with carbon and PVDF

We mixed 10 mg (14.3%) of Super P carbon black (IMERYS) and 60 mg (85.7%) of PVDF (KUREHA KF Polymer), and sonicated the solution using an ultrasonic cleaner (AS ONE US CLEANER US-2R) in NMP (FUJIFILM Wako Chemicals). The resistance was controlled by varying the amount of Super P carbon black and PVDF. PVDF is widely used as binder material in lithium-ion battery^36,37^. It has good adhesive property for carbon and silicone materials. This leads to electrical stability of the carbon material because PVDF prevents carbon nanoparticles from detaching from the surface of silicone^38^. The solution was poured into porous silicone. Conductive porous silicone was obtained after drying NMP for 12 h at 70 °C.

#### Low resistance paste with carbon

Super P carbon black (44 mg) was added into 1 ml chloroform (FUJIFILM Wako Chemicals). After dispersing using an ultrasonic stirrer for 30 min, this solution was mixed with Ecoflex 00-10 for 2 min at 2000 rpm, and air was removed for 1 min at 2,200 rpm using a rotation-and-revolution mixer. The Ecoflex containing Super P was cured at 70 °C for 15 min.

### Measurement of pressure and x-and y-strains sensing using a single-pixel device

Measurement of strain sensing was conducted using a tensile tester. The tester was connected to the carbon paste lines on the device. Furthermore, the tester was connected to column and row electrodes to measure the resistance variations of the pressure sensor. Pressure and x- and y-strain sensing measurements were conducted following the application of tension and pressure either simultaneously or alternately. The control system consisted of a switching integrated circuit and Arduino Uno switch circuits for resistance measurement with a millisecond temporal resolution.

### Stretchable pressure mapping device for 9-pixel pressure and x- and y-strain sensor measurements

In the 9-pixel mapping test, two sensors were pressed with application of uniform pressure when the device was subjected to strain. The resistance variations of all the sensing elements were measured. The pressure was 0.3 N and the strain was 20%. Electrical resistances were measured and calculated based on the theory introduced in the Supplementary Information section.

### Demonstration of a stretchable keyboard on a wrist to control electric display and sensed strain

Display control was demonstrated using the device after attaching it on the wrist. This displayed the word “MEMS” when the device was subjected to large deformations. In this demonstration, the response of the display equipped with a strain indicator was observed according to bending of the wrist and application of pressure. The five-level strain indicator was composed of yellow LEDs indicating the degree of the strain according to bending motion of the wrist. The first level of the LED indicated an 8% increase of the strain sensor. On the other hand, the LED monitor can display three types of characters depending on the pressure sensor that was pressed in the 2-pixel device. The letters “M,” “E,” and “S,” were displayed on a 16-segment LED display according to the combination of input signals from the two pressure sensors. As shown in Fig. 4b, the 16-segment LED display did not light up following the application of strain, even though the LED light bars were turned on. This is because the pressure sensor did not sense the strain during the bending process, although it can sense pressure effectively when it is pressed. Supplementary Figs. S16 and S17 show the circuit diagrams of the strain system indicator and 16-segment LED display.

## Supplementary information

Supplementary Figures

Supplementary Video S1
