# Supplementary material for: Highly stretchable sensing array for independent detection of pressure and strain exploiting structural and resistive control
Source: Sci Rep. 2020 Jul 29;10:12666. doi: 10.1038/s41598-020-69689-2 (PMC7391712; doi:10.1038/s41598-020-69689-2)
Supplement: Supplementary file 1 — Supplementary Figures [file 41598_2020_69689_MOESM1_ESM.pdf]

Supplementary Information

Highly Stretchable Sensing Array for Independent Detection of Pressure and Strain Exploiting Structural and Resistive Control

Ryosuke Matsuda[1], Satoru Mizuguchi[1], Fumika Nakamura[1], Takuma Endo[1], Yutaka Isoda[1], Go Inamori[1], and Hiroki Ota,[1,2]*

[1]Department of Mechanical Engineering, Yokohama National University, 79-5 Tokiwadai, Hodogaya-ku, Yokohama, 240-8501, Japan

[2]Graduate School of System Integration, Yokohama National University, 79-5 Tokiwadai, Hodogaya-ku, Yokohama 240-8501, Japan

*Corresponding author

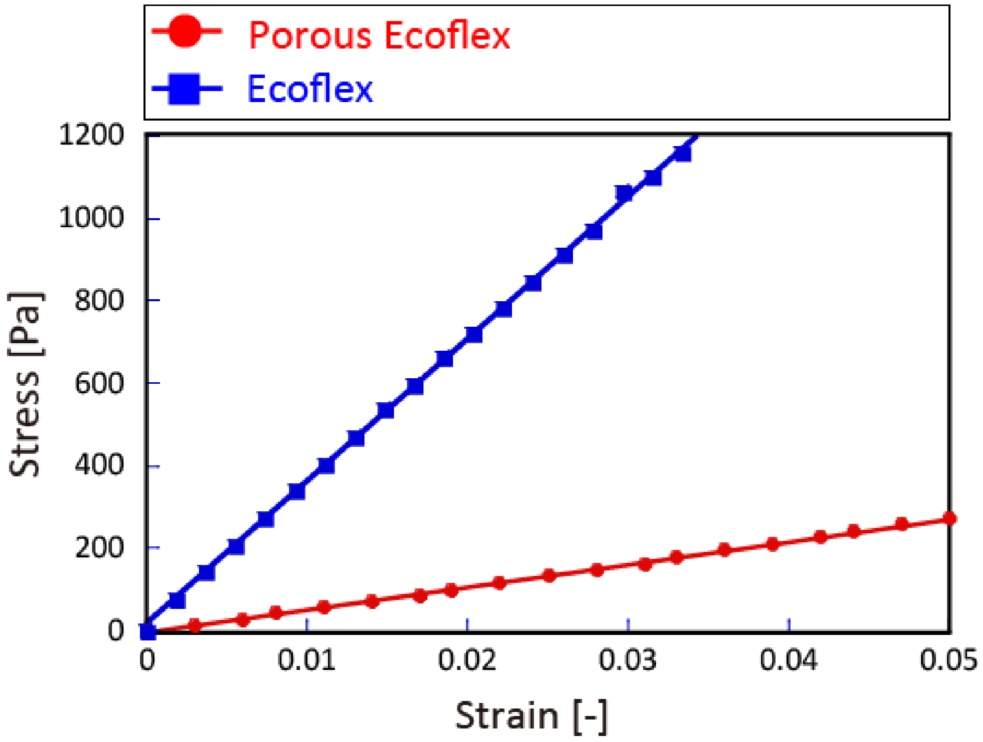

Supplementary Figure S1. Stress–strain relationship of porous silicone. The Young's moduli of porous silicone and regular Ecoflex were 5.50 kPa and 34.5 kPa, respectively.

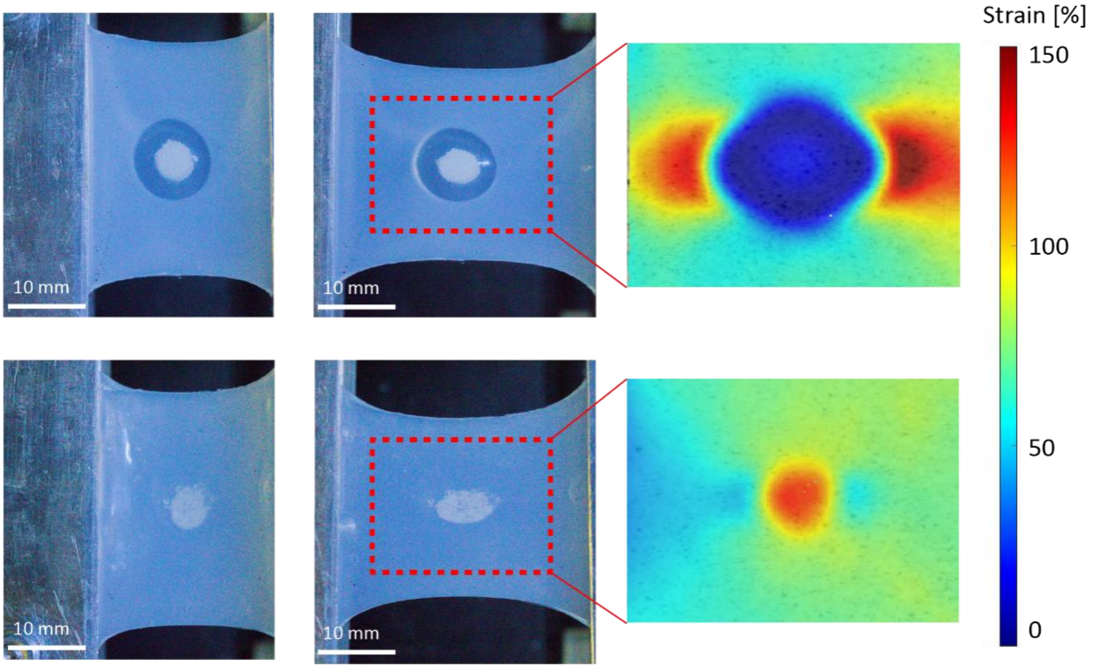

Supplementary Figure S2. Stability of pressure sensing elements during the application of 50% strain.

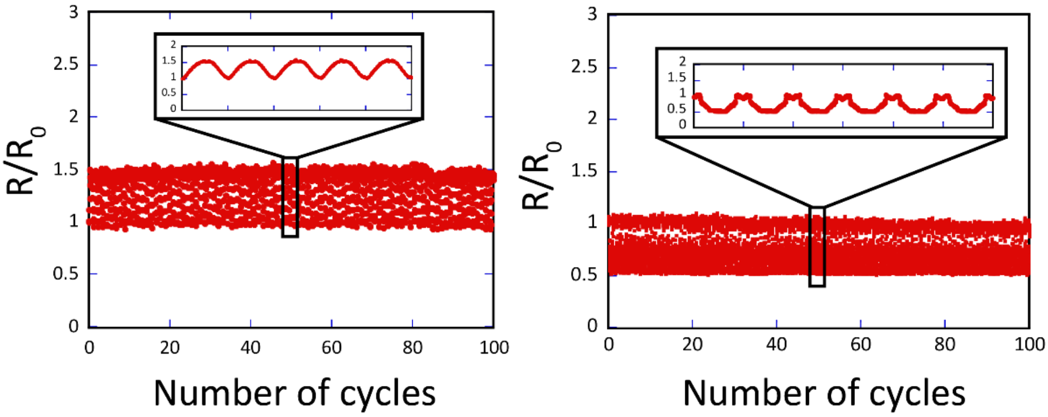

Supplementary Figure S3. Repeatability tests of pressure and strain sensors. In the strain tests, the device was stretched up to 50% strain 100 times, whereas in the pressure tests, a pressure of 15 kPa was applied on the device 100 times.

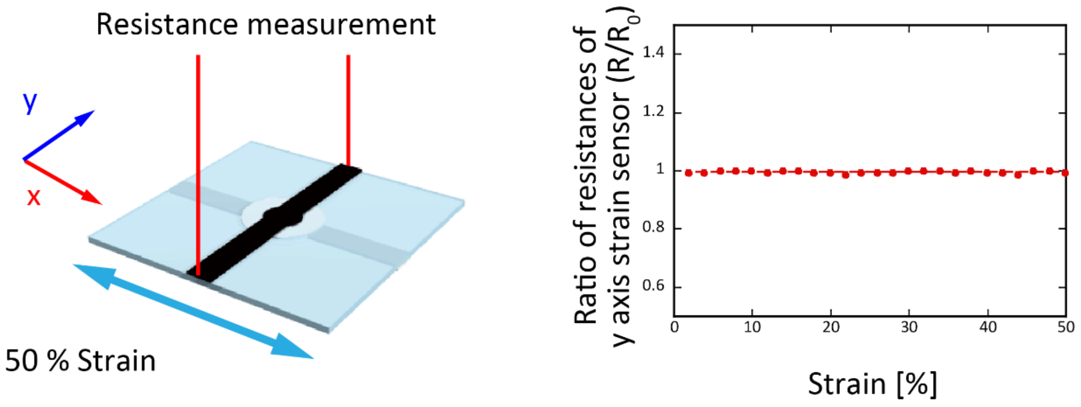

Supplementary Figure S4. Y-axis strain detection under x-axis tensile deformation.

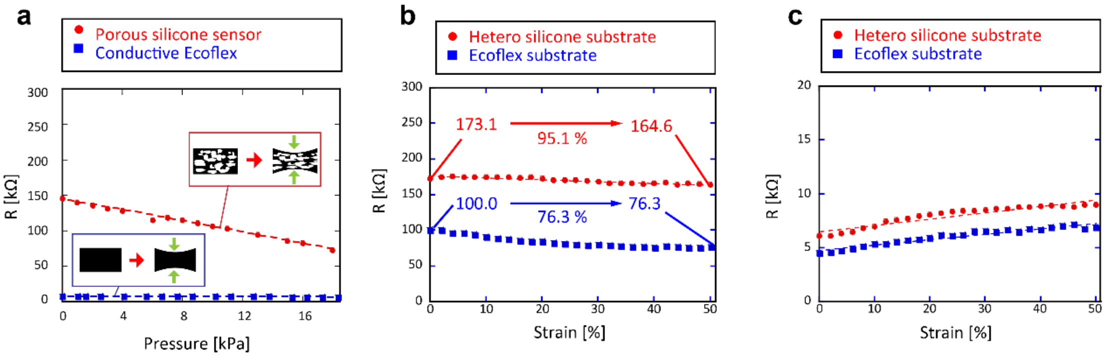

Supplementary Figure S5. Sensing characteristics of 1-pixel devices using the real resistance value.

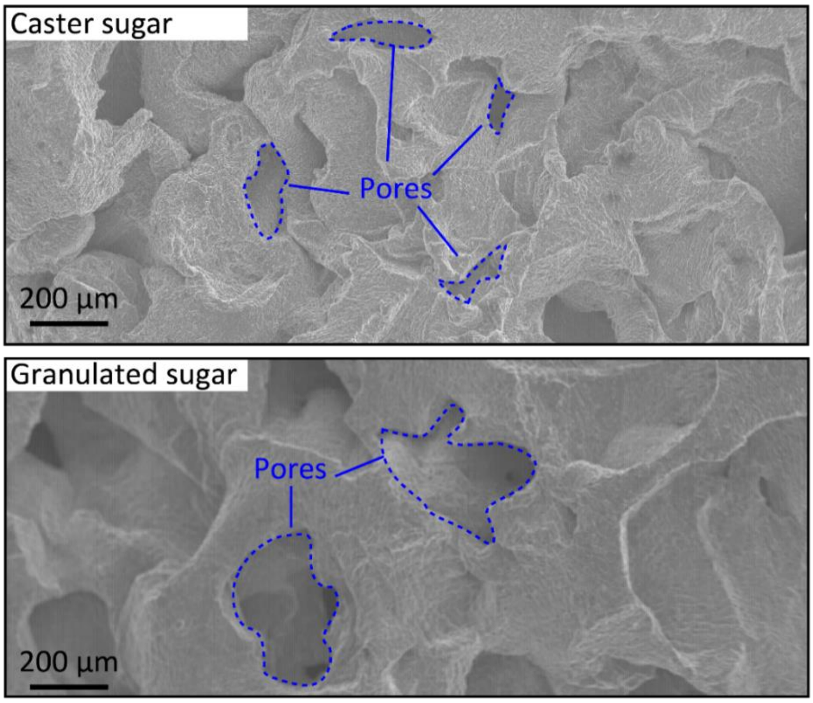

Supplementary Figure S6. Differences in pore size of caster and granulated sugar based on scanning electron microscopy images.

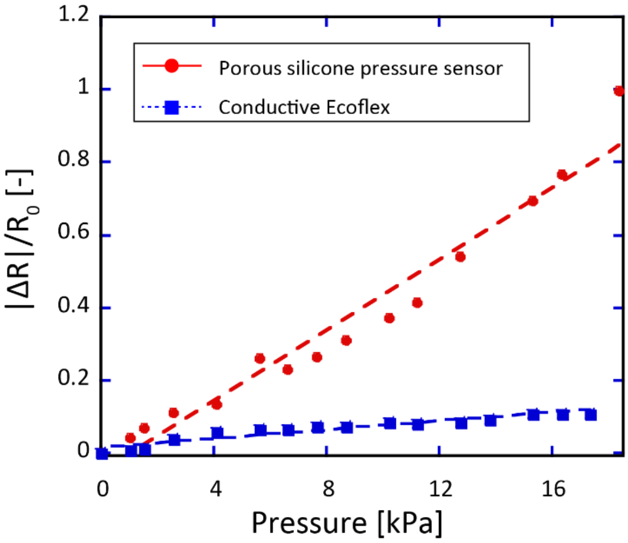

Supplementary Figure S7. Resistance variations of pressure sensors with respect to pressure. The sensitvity of the pressure sensor that used porous silicone (red) was 4.6% kPa^{-1}, and that of the pressure sensor that used regular Ecoflex and carbon particle mixture elastomer (blue) was 0.6% kPa^{-1}.

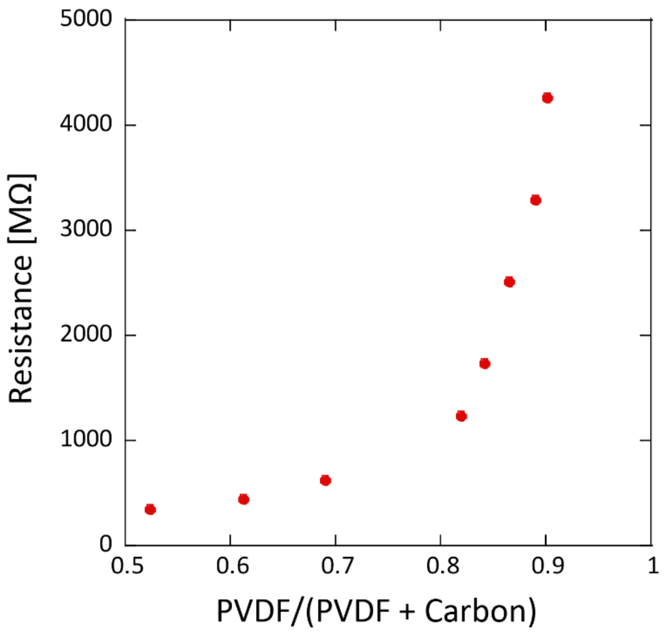

Supplementary Figure S8. Resistance variations with respect to the ratio of PVDF in the PVDF and carbon mixture. The resistance increases as a function of the amount of PVDF.

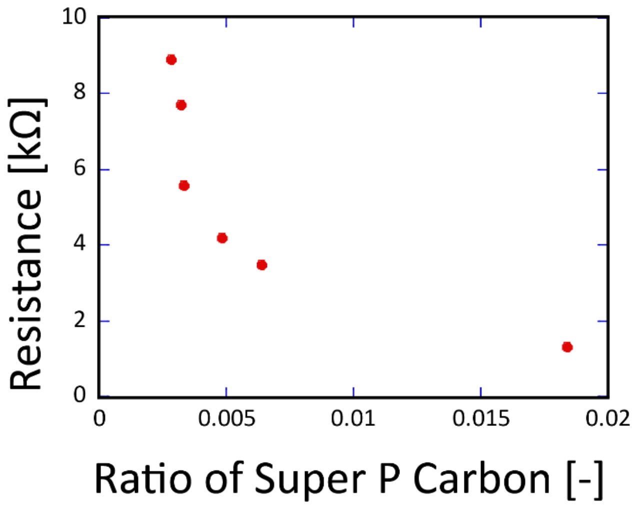

Supplementary Figure S9. Resistance variations with respect to the ratio of Super P carbon in porous silicone. The thickness of the porous silicone in this test device was 1.5 mm. The width and length were 5 mm and 30 mm, respectively.

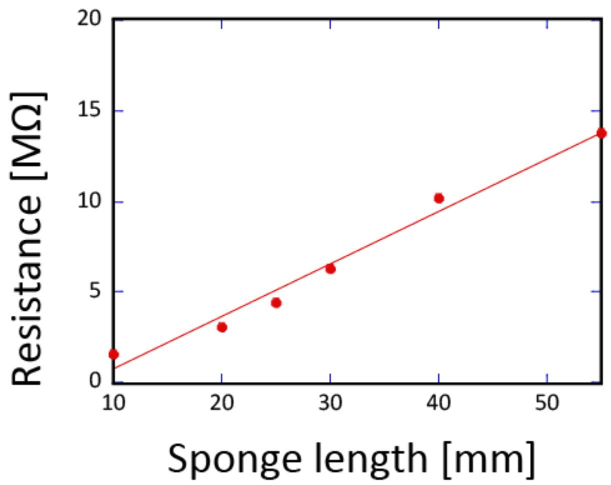

Supplementary Figure S10. Resistance variations with respect to the length of porous silicone electrodes coated with Super P carbon.

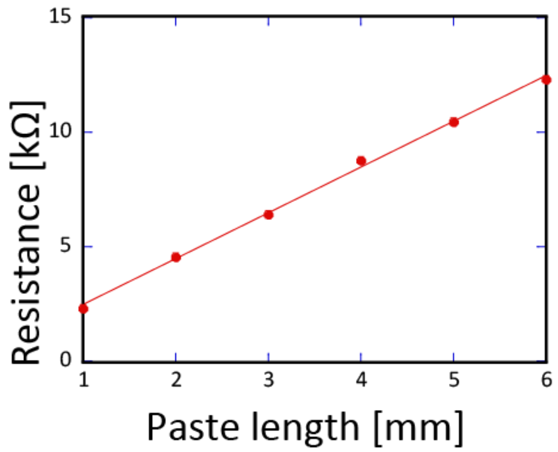

Supplementary Figure S11. Resistance variations with respect to the length of conductive silicone paste electrode.

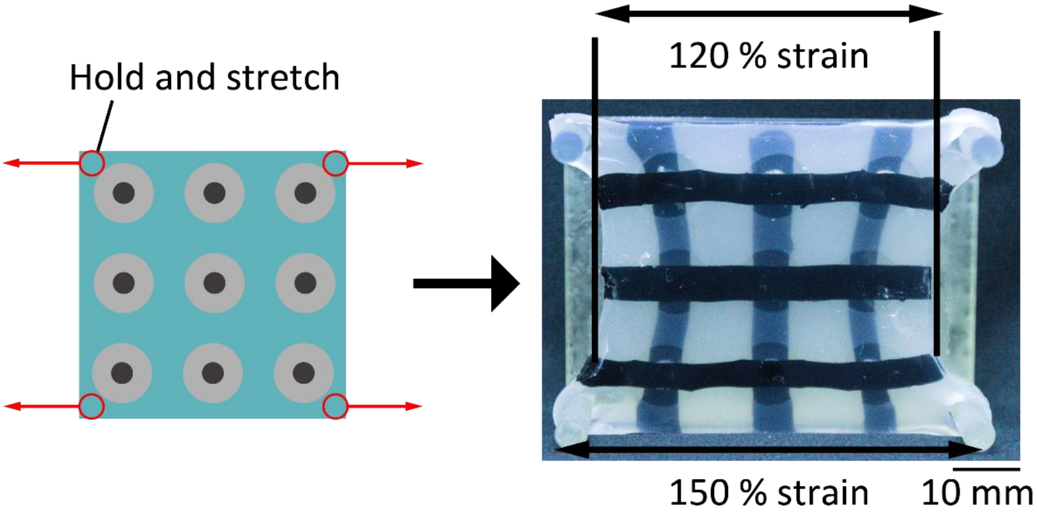

Supplementary Figure S12. Holding points of the 9-pixel strain test.

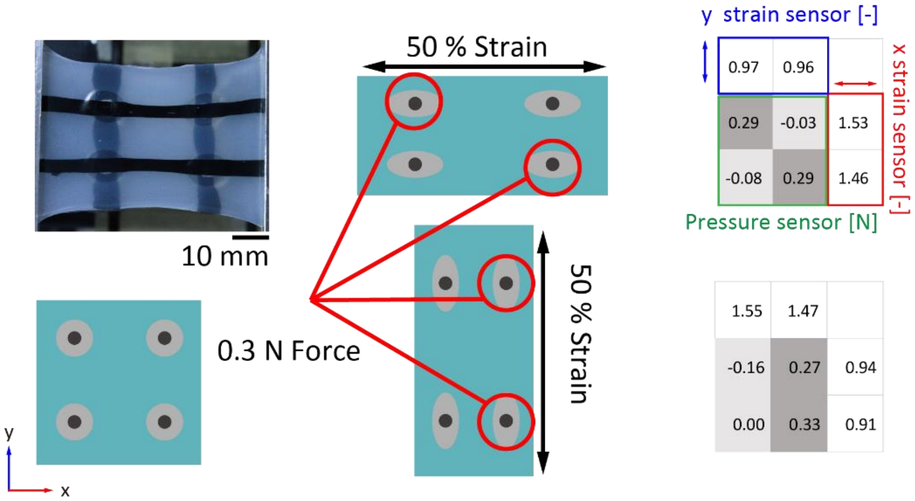

Supplementary Figure S13. Demonstration of the 4-pixel device.

Supplementary Table S1. Comparison of stretchable pressure sensors used in this study and other studies.

Reference	Method	Material	Pressure limit [kPa]	Sensitivity [% kPa^{-1}]	Strain limit [%]
i) Zhu et al.[18]	Resistive	Graphene	0 - 0.1	553	Not independent
			0.1 – 1.4	1	Not independent
ii) Choong et al.[34]	Resistive	Conductive polymer	0 - 3	1000	Not independent
			3 - 8	200	Not independent
iii) Gao et al.[27]	Resistive	Liquid metal	50	2	Not independent
iv) Lipomi et al.[23]	Capacitive	CNT + Ecoflex	800	0.023	Not independent
v) S. Yao and Y. Zhu[24]	Capacitive	Ag nano wire	0-400	0.162	Not independent
			400-1300	0.057	Not independent
vi)Y. Pang et al.[25]	Resistive	Graphene	1000	0.09	Not independent
vii) Park et al.[35]	Resistive	Gold film	1	200	15
viii) This study	Resistive	Carbon mixture	18	4.8	50

Supplementary Information 1
Measurements of 9-pixel pressure maps

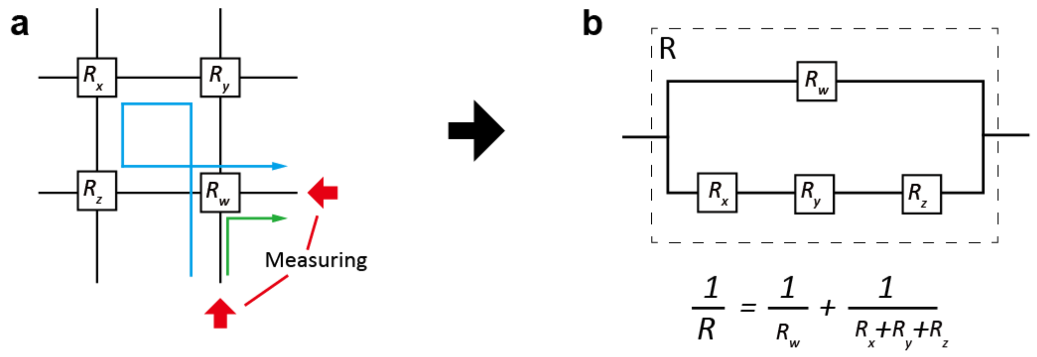

Supplementary Figure S14. Schematic diagram illustrating the measurement method with a 4-pixel device. a. Measurement points and electrical paths b. Combined resistance.

Supplementary Fig. S10 shows two electrical paths in the case of pressure sensing at point w using the 4-pixel device. All electrodes and sensor elements in the developed device are conductive. In addition, the sensing mechanism of the developed device was piezoresistive effect. This is the reason the actual resistance at point R_w is equivalent to the combined resistance (Equations 1 and 2):

$$\frac{1}{R} = \frac{1}{R_w} + \frac{1}{R_x + R_y + R_z} \tag{1}$$

$$R = \frac{R_w(R_x + R_y + R_z)}{R_x + R_y + R_z + R_w} \tag{2}$$

It is assumed that three resistance values, except the resistance of point w, decreased to 1/2 when pressure was applied at points x, y, and z. The combined resistance R′ obtained using the same two-point measurement method is as follows (Equations 3 and 4):

$$\frac{1}{R'} = \frac{1}{R_w} + \frac{1}{\frac{R_x}{2} + \frac{R_y}{2} + \frac{R_z}{2}} \tag{3}$$

$$R' = \frac{R_w(R_x + R_y + R_z)}{R_x + R_y + R_z + 2R_w} \tag{4}$$

Therefore, the combined resistance decreases even though point w was not pressed. Therefore, it is difficult to measure pressure values precisely using only the resistances of the column and row.

In this study, the following method was adopted for mapping measurements. The 9-pixel device in Supplementary Fig. S11 yields nine resistance values (R_r, R_s, R_t, R_u, R_v, R_w, R_x, R_y, and R_z) from the calculation of nine simultaneous equations based on nine combined resistance values (R_a, R_b, R_c, R_d, R_e, R_f, R_g, R_h, and R_j) (Supplementary Fig. S11 and Equations 5–13).

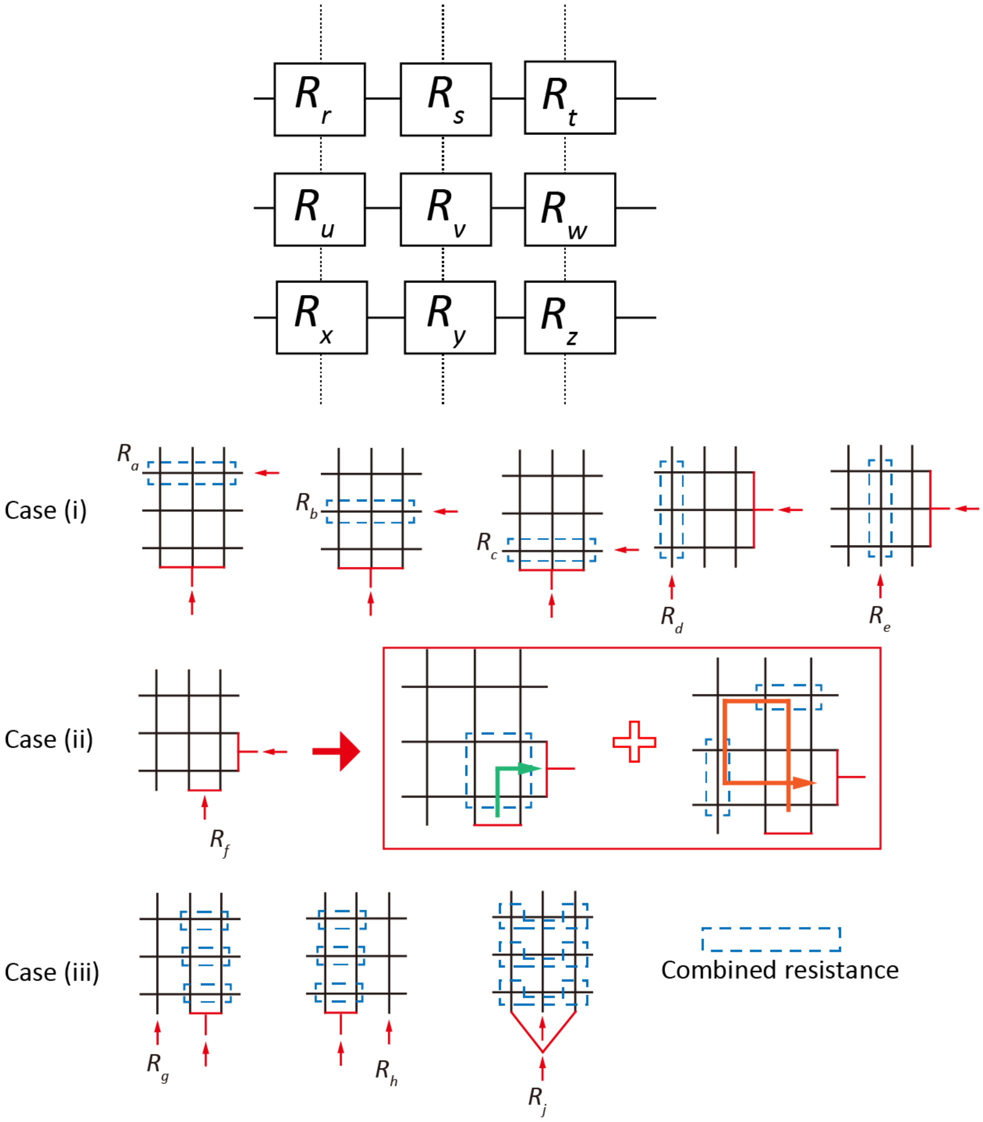

Supplementary Figure S15. Measurement method used in the 9-pixel device.

First, given that the three electrodes are connected (red line) and the resistances are measured in

accordance with the red arrows (case (i)), *Rr* can be measured. Using the same method, R_s, R_t, R_u, R_v, R_w, R_x, R_y, and R_z can be measured. Second, given that the two electrodes are connected and the resistances are measured in accordance with the red arrows (case (ii)), the combined resistance *Rf* can be measured. Finally, given that the two electrodes are connected (red lines) and the resistances are measured in accordance with the red arrows (case (iii)), the combined resistance *Rg* can be measured. Using the same method, *Rh* and *Rj* can be measured. According to the basic theories of electric circuit, the combined resistances are as follows (Equations 5–13):

$$\frac{1}{R_r}+\frac{1}{R_s}+\frac{1}{R_t}=\frac{1}{R_a} \tag{5}$$

$$\frac{1}{R_u}+\frac{1}{R_v}+\frac{1}{R_w}=\frac{1}{R_b} \tag{6}$$

$$\frac{1}{R_x}+\frac{1}{R_y}+\frac{1}{R_z}=\frac{1}{R_c} \tag{7}$$

$$\frac{1}{R_r}+\frac{1}{R_u}+\frac{1}{R_x}=\frac{1}{R_d} \tag{8}$$

$$\frac{1}{R_s}+\frac{1}{R_v}+\frac{1}{R_y}=\frac{1}{R_e} \tag{9}$$

$$\frac{1}{\frac{1}{\frac{1}{R_v}+\frac{1}{R_w}+\frac{1}{R_y}+\frac{1}{R_z}}}+\frac{1}{\frac{1}{\frac{1}{R_u}+\frac{1}{R_x}}+R_r+\frac{1}{\frac{1}{R_s}+\frac{1}{R_t}}}=\frac{1}{R_f} \tag{10}$$

$$\frac{1}{\frac{1}{R_x}+\frac{1}{R_y}+R_z}+\frac{1}{\frac{1}{R_u}+\frac{1}{R_v}+R_w}+\frac{1}{\frac{1}{R_r}+\frac{1}{R_s}+R_t}=\frac{1}{R_g} \tag{11}$$

$$\frac{1}{\frac{1}{R_y}+\frac{1}{R_z}+R_x}+\frac{1}{\frac{1}{R_v}+\frac{1}{R_w}+R_u}+\frac{1}{\frac{1}{R_s}+\frac{1}{R_t}+R_r}=\frac{1}{R_h} \tag{12}$$

$$\frac{1}{\frac{1}{R_x}+\frac{1}{R_z}+R_y}+\frac{1}{\frac{1}{R_u}+\frac{1}{R_w}+R_v}+\frac{1}{\frac{1}{R_r}+\frac{1}{R_t}+R_s}=\frac{1}{R_j} \tag{13}$$

Each resistance (R_r, R_s, R_t, R_u, R_v, R_w, R_x, R_y, and R_z) in the array pressure sensor can be calculated

by solving these simultaneous equations numerically using the Newton method.

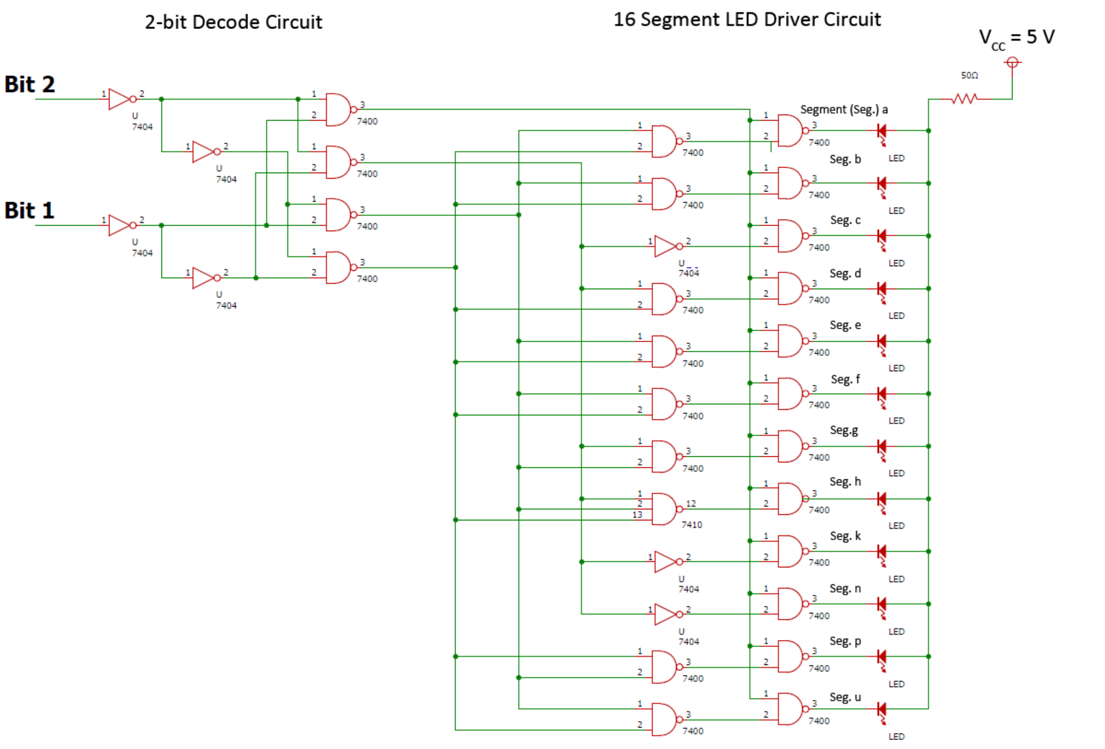

Supplementary Figure S16. Circuit diagram of a 2-pixel keyboard device.

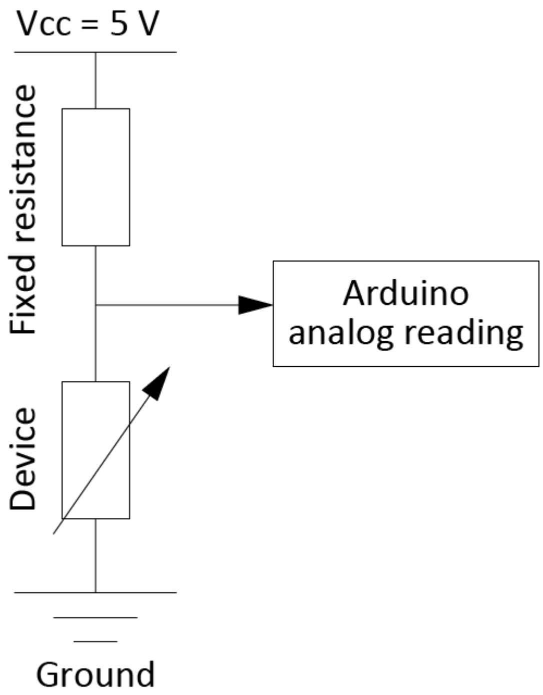

Supplementary Figure S17. Circuit diagram of the strain indicator.

Supplementary Video S1 demonstrates the display control with indicative strains of the device.
